# The Statistics of Computer Clocks and the Design of Synchronization Algorithms

**DOI:** 10.6028/jres.125.008

**Published:** 2020-02-25

**Authors:** Judah Levine

**Affiliations:** 1 Time and Frequency Division, National Institute of Standards and Technology, Boulder, CO 80305, USA

**Keywords:** computer clocks, frequency-lock loop, network time, phase-lock loop, synchronization algorithm, two-sample variance

## Abstract

In this study, I used standard statistical tools (such as the various forms of the two-sample Allan variance) to characterize the clocks in computers, and I show how the results of this study are used to design algorithms to synchronize the computer clocks. These synchronization algorithms can be used to synchronize the time of a computer to a local reference clock or to a remote server. The algorithms by themselves are not intended to be a simple replacement for software that implements the Network Time Protocol (NTP) or any other similar application. Instead, they describe the statistical principles that should be used to design an algorithm to synchronize any computer clock by using data from any external reference received in any format. These algorithms have been used to synchronize the clocks of the computers that support the Internet Time Service operated by the National Institute of Standards and Technology (NIST), and I illustrate the performance of the algorithm with real-time data from these servers. In addition to presenting the design principles of the algorithm, I illustrate the principles with two specific examples: synchronizing a computer clock to a local reference signal, and the design of a synchronization process that is based on time-difference data received from a remote server over the public Internet. The message exchange between the local system and the remote server in this configuration is realized in NTP format, but that is not a fundamental requirement.

## 1 Introduction

The time servers operated by the Time and Frequency Division of the National Institute of Standards and Technology (NIST) were initially synchronized by the LOCKCLOCK algorithm [1]. This algorithm used a combination of a frequency-lock loop to provide long-term stability and a phase-lock loop for short-term accuracy. (I use the term *accuracy* to mean the closeness of agreement between a measured quantity and a true value of the measurand. In the current context, the “true value” of the measurand is the value of Coordinated Universal Time [UTC(NIST)] realized by the NIST timescale. The determination of the accuracy of a measurement is limited by both systematic and random errors and cannot be completely specified by statistical considerations.) The implementation was designed to use periodic connections to the NIST Automated Computer Time Service (ACTS) [2]. The connections were implemented with standard modems connected to dial-up telephone circuits. The interval between calibration connections was chosen based on a comparison between the stability of the ACTS servers as seen through the dial-up telephone lines and the free-running stability of the computer clock oscillator. (I use the term *stability* to describe the variation of a measured quantity with time. This variation is characterized in statistical terms.) The accuracy of the synchronization process was defined by the symmetry of the delay through the telephone system. This accuracy varied somewhat from one circuit to another, but it was typically on the order of 0.001 s RMS, (root mean square) where the RMS average was estimated based on the performance of the 25 telephone circuits that were used to support the time service. The statistics of the controlled clock could be characterized as white phase noise. The magnitude of the Allan deviation was approximately 10^−3^/τ, where τ is the time between measurements, so that the accuracy and stability at 1 s were of comparable magnitudes.

The ACTS protocol is based on the usual two-way method in which the one-way transmission delay between the client and the server is estimated as one-half of the measured round-trip value. Both the accuracy and the stability of the time differences depend on the validity of the two-way method in estimating the one-way channel delay. The inbound and outbound delays will be equal when the connection is realized by a simple physical circuit, and this model was an accurate characterization of the dial-up telephone network in the 1980s, when the ACTS protocol was designed. The symmetry and stability of the time delay of dial-up telephone circuits were gradually degraded as the dial-up telephone system moved from a circuit-switched to a packet-switched configuration. Although it was possible to compensate for this degradation in the delay to some extent [3, 4], both the accuracy and the stability of the Internet Time Service transmissions were significantly degraded. The degradation at intermediate periods (averaging times of a few hundred seconds or less) was about a factor of 20, so that the accuracy of the time at the server was often no better than 15 ms to 20 ms, with a bimodal character that made averaging ineffective. As I will discuss in the next section, this level of performance was inadequate for the financial and commercial users of the NIST time service, who were required to use time stamps that were traceable to the NIST timescale. This paper discusses the new algorithm that was designed to mitigate this degradation and to provide support for the increasing number of financial and commercial users of the NIST Internet Time Service. The algorithm was based on an optimum combination of the statistics of the local clock oscillator and the statistics of the reference time signal, which may be received over a channel with time-varying statistics. The discussion is focused on the local clock and the channel that links the local clock to the reference time signal. The characteristics of the reference source are not discussed, and the details of the reference are not important. As I will discuss below, the reference clock is both much more stable and much more accurate than either the local clock or the channel that links the local clock to the reference. The synchronization information flows from the reference clock to the device under test through an imperfect, noisy channel, and no data flow in the reverse direction. The stochastic variation in these data are due to the statistics of the channel, the local clock, and the local measurement process, and the methods described here are designed to characterize and mitigate the impact of these effects.

I used the time deviation statistic (TDEV) in this study to characterize the clocks and the measurement process [5]. This statistic is also discussed in more detail in the Appendix. It is particularly useful for this study because it provides estimates of nonstationary noise processes, especially flicker noise, which makes an important contribution to the variance of the data, as I will show below. Although the causes of the variance are often understood in general terms, it is generally neither possible nor even necessary or useful to relate the variance to the variation in some specific physical parameter, especially since the parameter is often not under the control of the measurement process.

The algorithm described here is much more powerful and general than the LOCKCLOCK algorithm I described previously, which was limited to white-noise processes. In addition, it is much more sophisticated than the algorithms that are used in the public version of the Network Time Protocol (NTP) [6]. The combination of the local and remote data is periodically reevaluated in response to changing conditions, and it is designed to produce a clock that has better statistical properties than either contribution by itself. In addition, the algorithm characterizes the stability of the local clock oscillator, which is used to support holdover stability if the reference signal is not available for any reason. This is a significant advantage over simpler algorithms, which simply steer the local clock to the remote reference in a master-slave relationship. Finally, the algorithm can detect and compensate for nonstochastic variations in the frequency of the local clock oscillator, such as the variation that is often generated by the diurnal temperature fluctuations of the system environment.

The algorithm could be used to implement a time server or a time client, but it requires additional software to support either of these functions. In the NIST server implementation, the algorithm is used to synchronize the computer system clock by means of signals from a locally connected reference, and several independent processes respond to requests for time in a number of different formats with messages derived from the computer system clock. In the NIST client implementation, the algorithm is augmented by front-end software that exchanges messages with a remote system and passes the time-difference data back to the algorithm, which is an independent process. The front-end system uses the NTP format for the message exchanges in the example that I discuss, but that is not the central point of the discussion, and any message exchange format could equally well have been used. (The software that implements the message exchange is simply an interface between the source of the messages and the software that controls the clock synchronization. This separation of the data-receiver and data-analysis functions is used so that the synchronization algorithm can be flexible and does not need to be designed using the details of how the data are received.) The statistical characteristics of the link are important when the algorithm is used to synchronize the time of a client, as I will discuss below. In general, these statistical characteristics do not depend on the format of the messages that is used to provide the estimated time differences.

The new algorithm realizes more than an order of magnitude improvement in both the accuracy and the stability of the time of the computer clock relative to LOCKCLOCK [1], and it also includes a method to estimate the impact of temperature fluctuations on the frequency of the oscillator and similar effects that can be characterized by parameters other than a mean and a variance (such as a diurnal effect, for example). These frequency fluctuations are most important for averaging times longer than a few hours, but they have a measurable impact on the stability of the frequency even for shorter periods of a few thousand seconds. The process of estimating and removing these fluctuations therefore improves the stability of the oscillator at intermediate periods and results in a much greater improvement in the stability at longer periods, which determines the holdover performance of the system.

The design of the algorithm is based on the presence of an ensemble of cesium clocks at the location of each server. The local clock ensembles are synchronized to UTC (NIST), and these clocks provide the reference time for the servers; dial-up telephone connections to the ACTS system are no longer used.

Two methods are used to synchronize the local cesium clocks. The servers that are located at the NIST laboratories in Boulder are synchronized by a direct hardwired connection to the NIST primary timescale ensemble. This ensemble realizes UTC (NIST) by definition; the ensemble data are transmitted to the Bureau International des Poids et Mesures (BIPM) and contribute to the computation of International Atomic Time (TAI) and Coordinated Universal Time (UTC) by the BIPM [7]. The difference between UTC (NIST) and UTC is a slowly varying function of time with an amplitude of a few nanoseconds. This variation is negligible for purposes of the current discussion [8].

The cesium reference clocks at the other sites are compared to the primary NIST ensemble by using the global positioning system (GPS) common-view method [9], in which the remote sites and the NIST primary laboratory site measure the differences between the times transmitted by all of the satellites in view and the local clock. The differences among these data provide estimates of the times of the remote stations with respect to UTC (NIST). The accuracy of these time differences is typically not worse than 10 ns [10], which is also negligible for the current discussion.

The free-running frequency stability of the cesium ensembles at each of the sites is of order 2 × 10^−14^ for any averaging time longer than about 1 d, and the corresponding time dispersion is a few nanoseconds. Therefore, the local cesium clocks are more stable than the time received from the GPS satellites for periods less than a few days, and the GPS data are used primarily to validate the performance of the remote clock ensembles. These data could be used for steering the remote clocks to UTC (NIST) in principle, but this is seldom needed. (A steering adjustment may be needed about once or twice a year.) This independence is an important design advantage, since the performance of the system would not be degraded if the GPS signals were jammed, spoofed, or not available for any reason.

The algorithm has been implemented on the time servers that are currently used to support the NIST Internet Time Service. The systems are standard, unmodified, commercial servers; they have a standard-format serial port, 4 GB of memory, a 2 GHz processor, and a 1 GB standard twisted-pair network interface. Several different models are used to support the service, but the computer system clock characteristics are very similar on all of them. The newer systems are able to handle a larger number of time requests (up to 190 000 requests per second), but they are otherwise not significantly different from the older hardware.

The systems are dedicated to the time service, and all applications that do not directly support the time service have been disabled or removed. The systems have no graphical user interface, no web services, and no user accounts. The components of the operating system kernel that implements the phase-lock loop that is used to control the computer system clock by the standard NTP distribution have been removed or disabled.

It is difficult to compare the performance of the current algorithm with the time accuracy or stability that is realized with the public distribution of the NTP software [11], which can function as either a client or a server (or both simultaneously) because it is not clear what constitutes “typical data.” See Refs. [12] and [13] and Fig. 5 in Ref. [1]. However, at best, the public distribution of the NTP software realizes the performance of the method described here only in very special situations.

The applications that respond to requests for time stamps are dedicated to that function and have no role in synchronizing the computer system clock. The algorithm described in this text is implemented by an independent process, the sole function of which is to synchronize the computer system clock and monitor the time performance. (The public NTP software combines these two functions, and I suggest that this combination is not optimum because the requirements and assumptions of the synchronization and time-server tasks are very different.)

The next section presents the user requirements, which were a strong motivation for implementing the method described here; Sec. 3 provides a brief description of the how computers keep time; and the following sections describe the details of the algorithm, illustrated with data from the NIST servers.

## 2 User Requirements

The NIST time service has a large number of financial and commercial users, and the needs of these users provided a strong motivation for developing the algorithm described here. These users can be divided into two broad groups: (1) users who query the NIST time servers directly, often with queries and responses that include digital signatures, and sometimes with circuits that have a very symmetric delay and very low jitter, and (2) users who use signals from a global navigation satellite system, such as the GPS system, and then distribute this time to clients over an internal network that is also designed with a stable delay and low jitter. The GPS receiver serves as the source of the reference time in this implementation, and the other systems on the networks are time-service clients. The algorithm will provide the greatest benefits to users in these two classes. Although the NIST Internet Time Service receives several hundred thousand requests per second over the public Internet, the *accuracy* of the time service provided in this way may not be adequate for satisfying the proposed future requirements of these users. The algorithm that is described here can provide better time stability even in this configuration, since it combines the data received over the network with the statistics of the local clock. This combination will attenuate the rapid fluctuations in the stability of the network channel, because the local clock is more stable than the remote reference seen over the unstable channel. It also provides a holdover capability that is possible only when the statistics of the local clock are explicitly used by the algorithm. As I will discuss below, this algorithm can support the current requirements of financial and commercial users without special networks; this is often not possible with the standard distribution of the NTP software [11].

Financial transactions in the European Union are governed by the Markets in Financial Instruments Directive and Regulations MiFID II and MiFIR [14], which were effective starting on January 3, 2018. These regulations require a traceability to UTC with an accuracy that is no worse than 1 ms, and the accuracy is required to be 100 µs or better for low-latency and high-frequency algorithmic trading.

Financial transactions in the United States are governed by Financial Industry Regulatory Authority (FINRA) Rule 4590 [15], which requires a traceability to UTC (NIST) with an accuracy of 50 ms. Although the current public-access NIST time service can usually support this 50 ms requirement without the need for a specially configured channel between the clients and the server, many (most) financial institutions engage in international trading and so are bound by the European rules. (This level of performance depends on the details of the client system and the channel from the client back to the server. It is often realized but is not guaranteed.) Many in the user community also assume that the United States will adopt the European rules in the foreseeable future. As will be shown below, the algorithm described here can support an accuracy of 1 ms with respect to UTC (NIST), even when the connection between the client and the server is over the public Internet without any special circuit conditioning.

The algorithm might support the more stringent accuracy requirement of 100 µs for some network connections, but this level of performance would probably not be generally possible. However, the algorithm makes optimum use of the free-running stability of the local clock, especially when feed-forward compensation of nonstochastic effects is applied, as I discuss below, so that the accuracy requirement of 100 µs can be realized without the need for a full-time conditioned network; in fact, a message exchange every few thousand seconds would be adequate, and a continuous, full-time network connection would not be needed.

## 3 Computer Clocks

All computers have a memory register composed of contents that represent the time since some time origin. The time origin varies from one system to another; a value of 0 UTC on January 1, 1970 is typical. The memory register represents the time as seconds and fractions since the origin. An internal oscillator generates periodic system interrupts, and the operating system increments the value in the register by a constant value each time a hardware interrupt occurs. The constant is chosen to be equal to the nominal period of the oscillator. For example, if the oscillator generates hardware interrupts every microsecond, then the system adds 1 μs to the memory register each time an interrupt occurs.

Both the memory register that holds the current time and the constant value that is added on each interrupt can be modified under program control. The frequency of the physical oscillator is usually not adjustable by program control. Except during a cold start, it is generally bad practice to modify the time register directly because such an adjustment can have undesirable side effects, so that both phase-lock loops and frequency-lock loops typically modify the value added to the time register on each oscillator interrupt. Incrementing or decrementing this value increases or decreases the effective frequency of the time system. A time adjustment typically amortizes the adjustment as the maximum possible frequency adjustment over the necessary time interval, while a frequency adjustment typically applies a static change to the increment value. The total cumulative change that can be applied to the increment value corresponds to a fractional frequency adjustment of about ±3.8 × 10^−3^; in other words, it takes at least approximately 260 s to adjust the computer system clock by 1 s. (Although it is very unlikely, there would be no simple software fix for applying a larger frequency adjustment, should that become necessary. If a frequency adjustment of that magnitude is required, it may be an indication of a hardware failure.) In normal operation, typical static adjustments are much smaller and are generally on the order of 10^−5^, which corresponds to a frequency adjustment of about 0.9 s/d. Dynamic adjustments are typically about a factor of 10 or 20 smaller than this value.

## 4 Time-Difference Measurements

The measurement of the time difference between the computer system clock and an external reference is the basis of any synchronization algorithm. In the algorithm that I am describing, the measurements are divided into two parts: a high-accuracy measurement that measures the fraction of a second with a resolution of nanoseconds, and a lower-accuracy measurement that identifies the correct integer second.

**Fig. 1:**
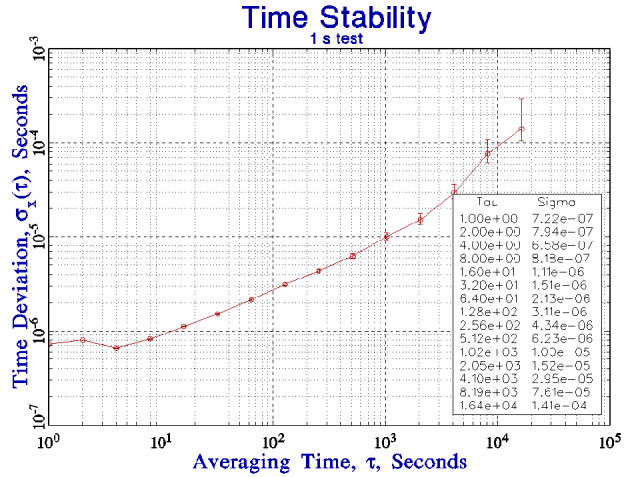
The time deviation of the measured time differences between the computer clock and an external reference. The variation in the time of the external reference is negligibly small on this scale.

The high-accuracy measurement is implemented by connecting a physical 1 Hz pulse to one of the interrupt lines of the system serial port. (The concept of time-difference data derived from a 1 pps signal [a pulse in which the leading edge is synchronized to the UTC second, where pps represents pulse per second] connected to one of the interrupt lines of a serial port is not new and is not the primary focus of this discussion. For example, the publicly available distribution of the NTP software has had this capability for some time [16, 17], but the algorithm described here that is used to discipline the computer system clock is not part of NTP.) The serial port driver is modified so that the system time is cached each time an external pulse is received. Since this pulse is generated by an external reference system that is locked to UTC (NIST), the value of the seconds-fraction portion of the system time register specifies the time difference (modulo 1 s) between the computer system clock and the external reference. Since the seconds-fraction register is always positive or zero, a value greater than 0 s and less than 0.5 s is taken to indicate that the computer system clock is fast with respect to the external reference, and a value greater than or equal to 0.5 s is taken to indicate that the computer system clock is slow. The jitter and the static latency (the minimum, load-independent time that elapses from the time when a pulse is received by the serial port hardware and the time when the application software is notified of that occurrence) in this measurement process are carefully controlled and are typically of order 0.8 μs RMS. (The jitter in the measurement process is the primary contributor to the TDEV at the shortest averaging times shown in Fig. 1.) As a practical matter, it is not possible to separate the noise in the measurement process from the phase noise of the local clock oscillator, and this value is a combination of both contributions, as I will discuss in the next section. The noise contribution of the measurement process is a function of the hardware, and it increases somewhat if the system is heavily loaded. The latency and jitter increase to about 3 μs RMS when the system receives more than about 190 000 requests per second for time in NTP format, and NIST currently has one server that is operating near this limit. The number of requests is modeled as satisfying Poisson statistics, so that fluctuations in the instantaneous count rate would be unlikely to be greater than ±900 requests per second (2σ). The observed short-term variation, based on ten 5 min samples in the number of requests, was +1000/−0 requests per second. This variation is asymmetric and is somewhat larger than would be expected based on simple Poisson statistics, but it is unlikely to produce a detectable change in the accuracy or stability of the time service. These effects are a function of the measurement process and are independent of the interval between measurements.

The lower-accuracy measurement process is typically implemented by an independent channel. The accuracy and jitter of this process only need to be small enough to unambiguously verify that the computer system clock is on the correct integer second. These data can be acquired through a serial port connection to an external clock or to a network connection to an external reference. These measurements do not need to be repeated very often after the initial cold start, and subsequent measurements are used primarily to verify that the clock in the time server has not jumped an integer number of seconds—an event that has never happened in normal operation.

## 5 Free-Running Stability of the Computer System Clock

The next step in the design of the synchronization algorithm is a measurement of the free-running stability of the computer clock. Figure 1 shows the time deviation (TDEV) of the time differences between the external reference signal and the free-running computer clock. These data were acquired as described in the previous section on a system that had no other processes active. The external reference signal was derived from an ensemble of cesium standards, so that the jitter in the reference time is negligible in this configuration. There are no missing data in this simple configuration, and the measured values for TDEV at the shortest averaging times (and especially the fact that it is dominated by flicker of phase noise) are consistent with the characteristics of a quartz-crystal hardware oscillator and the jitter in the software latency of the system that was used for these tests. This supports the assertion that these data are not degraded by statistically significant outliers. Flicker of phase noise is independent of the time interval between measurements, so that the TDEV values for averaging times greater than about 10 s, which increase approximately proportional to the square root of the averaging time, are due to the frequency fluctuations in the clock oscillator (Appendix; Table 1). This is an important conclusion. To the extent that the short-period fluctuations are due primarily to flicker of phase noise, an algorithm that attempts to attenuate these short-period fluctuations by adjusting the frequency of the clock oscillator is unlikely to improve matters and may make things worse because the variations in the oscillator frequency are not the source of the problem.

This conclusion is strictly true if the short-term noise can be characterized as white phase noise, since there is no correlation between the noise contributions for consecutive time-difference measurements in this case. As I will show below, this characterization is not true for the clocks in many computer systems, which are dominated by flicker of phase noise at short averaging times, so that adjustments to the frequency of the clock oscillator can improve its time stability for short averaging times to some extent.

The TDEV data can be used to identify three noise domains. The dominant noise type is flicker of phase for averaging times up to about 10 s; it is white frequency noise for averaging times from about 10 s to 10^3^ s; and it is flicker of frequency noise for averaging times greater than 10^3^ s (see Appendix; Table 1). The statistics shown in Fig. 1 are characteristics of the computer hardware that was used for this study, and they represent a benchmark against which our synchronization algorithms will be compared. In the subsequent discussion, these characteristics will be assumed to be independent of time and of any other process that is running on the system. The validity of this assumption was confirmed by characterizing the performance of heavily loaded production time servers, which use the same hardware and which were synchronized by using the method described here.

## 6 Synchronization Algorithm

The first step in the algorithm is to adjust the computer system clock so that it is nominally on time. To perform this adjustment, the algorithm uses the measured time differences, averaged over some interval, and applies a frequency offset to the clock oscillator to drive these differences to zero. (The exact value of the averaging interval is not important at this stage, since the time differences are larger than the measurement noise so that a coarse adjustment procedure is adequate. The primary purpose of the averaging is to detect glitches as I will describe below.) Removing a large time offset with a frequency adjustment can be a very slow process, so the algorithm steps the clock by modifying the time registers directly if the initial time difference is more than ±1 s. The frequency adjustment method is then used to remove the remaining time offset by applying the maximum possible frequency offset. As described earlier, this adjustment period will last no longer than 260 s.

An important consideration in the design of this process is ensuring that the control parameter is not influenced by outliers, including very large time-difference measurements that are not consistent with the previous estimate of the time difference, the frequency offset that has been previously applied, and the statistics of the clock as shown in Fig. 1. (Recall the statement above that the data in Fig. 1 are characteristics of the system and do not depend on time or on system load. Therefore, the detection of an outlier is based on a metric that was estimated independently of the data that are being evaluated at this point in the algorithm. This is an important advantage that is not always available.) Any method that is used to detect and reject outliers must be subjective to some extent, since there is no objective way of distinguishing between a measurement that is a glitch that should be ignored and a data point that is far from the expected value but nevertheless has a very small (but nonzero) probability of being consistent with the assumed distribution function, possibly with an increased standard deviation. Therefore, the method I discuss, which treats the glitch as a single-point, one-time, pure measurement error with no change in the underlying characteristics of the clock oscillator, is based more on experience than on a rigorous statistical justification. The value of the standard deviation that is used in the test described below is not important; as will be shown below, most (but not all) data either pass the test easily or fail to pass by a large margin This is not unusual for a flicker-noise measurement process. The performance of the algorithm is not sensitive to the value of the standard deviation that is used to detect outliers. In addition, the standard deviation of a flicker process depends on the precise data set that is used to computed it, and subsets of the data may suggest values for the standard deviation that are very different from the data set taken as a whole.

The algorithm first acquires five time-difference measurements in consecutive seconds, and then it arranges them in order from the largest value, *x*_5_, to smallest value, *x*_1_. (The choice of five values is somewhat arbitrary, and it was chosen primarily so that a minimum of three values will always be available for the majority voting method describe below. Increasing the number of data points increases the number of computer cycles that are required without necessarily suppressing the jitter, which arises from flicker-noise statistics.) If *x*_5_ − *x*_1_ < 3*σ_x_*(τ = 1 s), where *σ_x_* is a constant value taken from Fig. 1, then all of the measurements are consistent with the expected time deviation. In this case, accept all of the measurements, and compute the mean value in the usual way. Note that the data are not characterized as white phase noise, so that the mean depends on the data points that are used to compute it and the number of points that are used to form the estimate. The standard deviation of the mean is generally not a statistically robust quantity.

If the test in the previous paragraph fails, then compute *A* = *x*_5_ − *x*_4_ and *B* = *x*_2_ − *x*_1_. If *A* > *B*, then reject *x*_5_; if *B* > *A*, then reject *x*_1_. (In the unlikely event that *A* = *B*, then reject both the largest and smallest values and proceed to the three-point test described below.) Then, repeat the previous comparison with the remaining four data points. If the comparison passes, then compute the mean of the four points; if it fails, use the same method to compute the outlier, drop it, and repeat with three points. If the test passes, accept the mean of the three points; if the test still fails, reject all of the data and start again. Signal a fatal error if the three-point test fails a second time with different data.

An important aspect of this procedure is that it can be realized without multiplications or divisions until the last step, which computes the mean. This is important on a heavily loaded system, where computer cycles are at a premium. The algorithm is simplified somewhat on a system that is receiving more than 150 000 time requests per second because it is not fast enough in this situation on the hardware that is currently in use. The simplified algorithm immediately discards the largest and smallest measured time differences and either accepts or rejects the mean of the remaining three values based on the difference between the largest and smallest values as described above. In general, this comparison can be performed on the fractional-seconds portion of the system time by using integer arithmetic and without involving the integer-second portion of the computer system clock.

The measured time differences are not corrected for the time dispersion due to the frequency offset of the local clock. The mean of the measurements is assigned a time tag of the time associated with the midpoint of the surviving data, and this automatically compensates for the rate offset, assuming that it is a constant linear value over the measurement interval. (Depending on which points survive the previous tests, the time tag associated with the group may or may not be the time tag associated with one of the measurements. It must be explicitly computed if only four points survive or if the three points that survive do not have consecutive time tags.)

The result of this process is an estimated time difference of *δx*. If

$\left|\delta x\right|>3\sigma_{x}\left(\tau =1\;s\right)$ (1)

where *σ_x_* is the value from Fig. 1, then the clock is still in time-adjustment mode. Apply a frequency offset to amortize this time difference in *T* seconds:

$\delta f=-\frac{\delta x}{T}$ (2)

where *T* is calculated to guarantee that the adjustment will be completed before the next measurement cycle. If the magnitude of the time difference is large, then the frequency offset may be limited to the maximum value as discussed above. If the time-difference data are acquired every second, which is the usual case, then the time offset is amortized at a maximum rate of about 3.8 ms per measurement cycle. The parameter *σ_x_* in Eq. (1) is not the traditional standard deviation because it is estimated from data that are not characterized by a Gaussian distribution for any averaging time. Therefore, the 3σ rejection threshold in Eq. (1) should be taken as a reasonable value that is not rigorously justified by reference to the probabilities derived for a Gaussian distribution. This test is commonly used in the literature [18, 19], even for data that do not satisfy a Gaussian distribution.

As a specific example, assume that the time difference in Eq. (1) is such that the inequality in that equation is satisfied. The initial procedure has adjusted the time of the clock so that the magnitude of its difference is not greater than 1 s. Based on the values in Fig. 1, the magnitude of the time difference is less than 1 s but greater than about 2.5 µs. Apply a frequency correction to the system clock oscillator to drive this time difference to zero. The frequency correction is either the value given by Eq. (2) with *T* = 1 s or the maximum possible frequency correction described above, which will amortize the time difference by 3.8 ms. Continue in this mode, acquiring new data points for each cycle, until the inequality of Eq. (1) is no longer satisfied. Since the cold-start procedure set the clock to have a time offset that is not greater than ±1 s, this adjustment process is guaranteed to finish in no more than fifty-two 5 s cycles, *i.e*., somewhat more than 4 min.

A pure phase-lock loop method based only on the time-difference measurements could be used to control the frequency of the computer system clock even when the measured time differences are smaller than the value given by Eq. (1). Specifically, the value calculated by Eq. (2) would be applied to the clock on every measurement cycle to drive the time difference to zero before the start of the next cycle This strategy by itself is not optimum for a number of reasons. In the first place, the time differences at short averaging times are heavily contaminated by measurement noise, so that attempting to correct these differences by adjusting the oscillator frequency will not work as well as intended. (The time-difference data have already been averaged by the multipoint method described in the previous paragraphs, and additional averaging at this point will not improve matters significantly, especially because the domain in which the averaging of time differences is a reasonable strategy is limited to about 10 s, based on the results shown in Fig. 1. The multipoint method describe above uses about one half of this interval.) Second, the statistics of the measured time differences are not characterized as white phase noise, even for the shortest averaging times, so that the average of the measured time differences is not an unbiased, statistically robust estimator. (The average of time differences characterized as a flicker process, as shown by Fig. 1, is not a statistically robust estimator either of the current time difference or of the time difference at some future time.) Finally, this type of loop provides no information on the frequency accuracy or the stability of the computer clock oscillator. The lack of knowledge of the statistics of the local clock has two consequences. In the first place, there is no way to validate the accuracy of the time correction specified in Eq. (1), so a failure of the reference time system cannot be detected, including the channel that links it to the device under test. (The outlier detection process detects only short-term stability, so that a single-point large time difference generally will be detected and removed by the multipoint method described above. However, a large time difference that passes the multipoint test is a sign of a problem. The actual algorithm sets the time status to be unsynchronized and calls for help when this happens because there is generally no unambiguous method for deciding which system is at fault. This is treated as a failure that cannot be unambiguously repaired inside of the algorithm.) In addition, this method has no ability to provide some level of holdover performance should the connection to the external reference clock fail for any reason. I will discuss the question of holdover below. Therefore, the algorithm switches to a frequency-control mode when the time differences are reduced to a value smaller than the value given by Eq. (1). The algorithm almost never switches out of the frequency-control mode (<<1%) when the reference time signal is a local 1 pps signal. This is not always true when the reference time is received over a network with an unstable delay, as I will discuss in Sec. 7.

The frequency-control mode operates in the domain where the time differences are dominated by the white frequency noise of the clock oscillator. Based on the values in Fig. 1, this domain extends from an averaging time of about 10 s to an averaging time of about 10^3^ s (Appendix; Table 1). If *T*_min_ and *T*_max_ are the minimum and maximum averaging times of the white frequency domain, and if *δx* is the time difference estimated as described in the previous section, then the average frequency offset of the computer clock over the time interval from *t* − *T*_min_ to *t* can be estimated by

$y\left(t\right)=\frac{\delta x\left(t\right)-\delta x\left(t-T_{min}\right)}{T_{min}}$ (3)

where the first term in the numerator of Eq. (3) is an estimate of the time difference that was just acquired, and the second term is the corresponding estimate on the previous cycle at time *t* − *T*_min_. It is important to emphasize that *T*_min_ must be chosen to be long enough so that the time-independent measurement noise makes a small contribution to the measured time differences relative to the frequency noise of the clock oscillator. Also note that the time-difference measurement on any cycle implicitly includes the impact of all of the frequency adjustments that have preceded it. The implementation described here uses a measurement interval of about 200 s to satisfy this requirement. The current estimate of the frequency offset is then computed recursively based on the offset computed during the previous measurement cycle, *y*(*t* − *T*_min_) and the current offset estimated by Eq. (3) with a gain factor of *k*, given by

$k=\frac{T_{max}}{T_{min}}\;$(4)

so that

$\overset{-}{y}\left(t\right)=\frac{y\left(t\right)+ky\left(t-T_{min}\right)}{k+1}\;$(5)

and the frequency offset applied to the computer clock is the negative of the value in Eq. (5). In this mode, the primary control of the computer system clock is a frequency loop driven by the average frequency computed by Eq. (5), and where the input to the average in Eq. (5) is the frequency estimated by Eq. (3), which is based on the evolution of the time difference over the averaging time *T*_min_. The magnitude of this frequency offset has a nominally constant value that changes very slowly with time as a result of the aging of the quartz-crystal frequency reference. There is also a smaller and more rapid frequency variation due to the residual white frequency fluctuations, which are attenuated by Eq. (5), but are not completely removed. Finally, there are frequency fluctuations at intermediate periods that are probably driven by environmental perturbations, especially fluctuations in the ambient temperature.

The operation of the frequency-control loop is initiated only when the time difference has been reduced to a value that is close to zero statistically, as defined by Eq. (1), but a control loop based purely on frequency will also be stable when the time difference differs from zero by any constant value. Since *δx*(*t*) is very small in the frequency-control mode, the simplest solution is to apply a time step that sets the time difference to zero on every measurement cycle. This is implemented as an additional frequency offset on each measurement cycle. This additional frequency offset decreases the small-signal transient response. The magnitude of this additional frequency offset is not greater than approximately 3 µs/200 s = 1.5 × 10^−8^ (see Fig. 1); this value is algebraically added to the frequency computed in Eq. (5), and the result is then applied to adjust the frequency of the clock oscillator. This additional frequency correction simplifies Eq. (3), since its application has driven *δx*(*t* − *T*_min_) to 0. In the simple, ideal case, where there is no noise in the frequency of the oscillator or the measurement process, the frequency estimated by Eq. (5) will exponentially converge to the true frequency offset of the clock with a time constant of *kT*_min_, and the time difference will be driven to 0. The control loop operates in the domain where the variance of the time differences is dominated by white frequency noise, so that the estimate in Eq. (5) must provide the primary steering adjustment, and the control loop can become unstable if this condition is not enforced.

The results shown here are based on a value of *k* = 1000/200 = 5; the choice of the value for *k* must be based on a number of considerations. From Eq. (5), the frequency gain of the algorithm, *i.e*., the contribution of the current offset frequency estimate, Eq. (3), to the steering frequency estimated in Eq. (5), is approximately 1/(*k* + 1). If the simplifying assumption is made that the noise processes can be completely specified by Gaussian distributions, a pole-placement analysis can be used to estimate the time constant of the control process described by Eq. (5) [20, 21] when the system is critically damped. The time constant, *T*_c_, is approximately

$T_{c}=\frac{-2T_{min}}{ln\left(1-\frac{1}{k+1}\right)}≅11T_{min}=2200\;s$ (6)

The statistics of computer clocks do not satisfy the assumptions that are the basis of a pole-placement analysis, so that this estimate provides only approximate guidance on the performance of the actual system. I measured a step response of about 2000 s on the test system, but the noise processes are not stationary; the actual step response is reduced by the time steps described in the preceding paragraph and is also sensitive to the measurement noise when the test is performed. The agreement to within about 10% may be fortuitous.

The value of *T*_max_, the upper end of the averaging domain where the variance is characterized as white frequency noise, is sensitive to various environmental factors, such as the variations in the ambient temperature (see below). The noise of the measurement process compared to the frequency noise of the clock oscillator determines *T*_min_, the minimum averaging time where the variance of the time-difference data is dominated by the white frequency noise of the clock oscillator. Therefore, other things being equal, reducing the measurement noise would be very helpful, since it would increase the range of the white frequency noise domain and therefore support a larger value for *k.* The value of *T*_min_ varies somewhat from one system to another and also changes slowly as the oscillator hardware ages. Therefore, the parameters of the control algorithm are automatically reevaluated every few weeks.

Although it would be possible to increase the attenuation of the contribution of white frequency noise to the variance by increasing the value of the averaging time constant, *k*, this would come at the expense of an increase in the time constant of the control loop, which would result in decreased sensitivity to any intermediate-period contribution to the variance that could not be characterized as a white frequency noise process. The servers are typically operated in locations that have only modest environmental controls, so that these effects are almost always present.

The nearly diurnal fluctuations in the environmental temperature are an example of such a process, but there are others, such as fluctuations in the mains voltage, which can be approximately characterized as “bright lines” in the Fourier domain with nonstationary amplitudes and phases, rather than as one of the noise types that typically characterize crystal oscillators (Appendix; Table 1).

It is not practical to measure these extraneous inputs and compensate for them in some way. For example, the temperature-sensitive components of a computer are not known, and they may not even be localized to one area of the motherboard. In addition, there is no *a priori* estimate of the admittance. The method I discuss below estimates and removes the contributions of these extraneous effects without the need for an unknown number of ancillary measurements or knowledge of the relationship between the external stimulus and the resulting frequency variation.

**Fig. 2:**
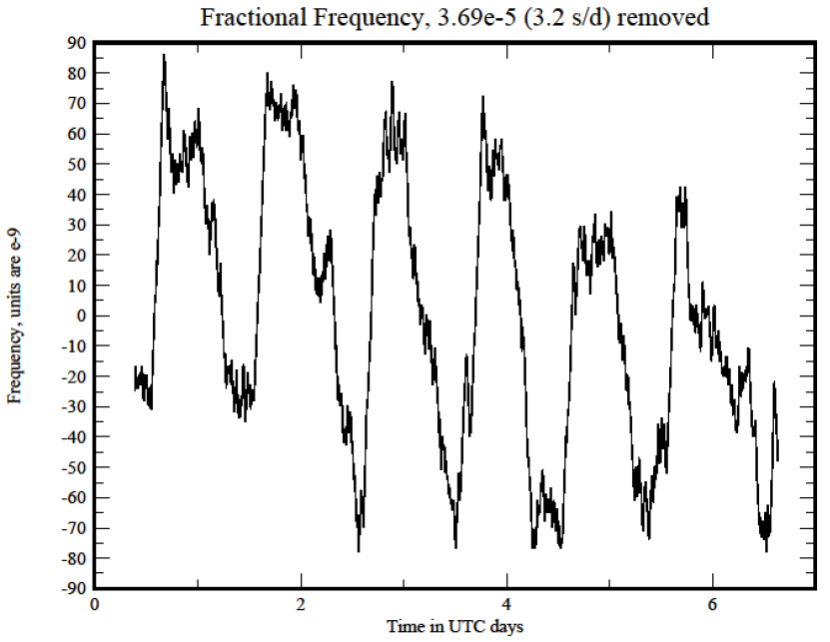
The offset frequency of the computer clock oscillator estimated by Eq. (5). The figure shows the nearly diurnal frequency variation of a free-running clock oscillator after a constant frequency offset has been removed, and the smaller and more rapid residual white frequency noise contribution. The white frequency contribution is attenuated by Eq. (5) but not completely removed. The diurnal variation is probably driven by changes in the ambient temperature.

An example is shown in Fig. 2, which plots the frequency estimated by Eq. (5) as a function of time. The plot shows the diurnal variation in the frequency estimate, which has a peak-to-peak amplitude of approximately 2 × 10^−7^, superimposed on a much larger, nearly constant frequency offset of 3.7 × 10^−5^. Note that many of the rapid changes in the frequency estimate occur near 0 UTC, which was 6 p.m.

(1800 h) local time when the tests were performed. The laboratory cooling system changes from day mode to night mode at this time, so that these frequency changes are almost certainly driven by changes in the ambient temperature. The identification of this relationship is reasonable, but it is neither necessary nor sufficient for this discussion, which treats the data from a statistical perspective without regard to any explicit stimulus. I will discuss these data in more detail in the section on holdover below.

**Fig. 3:**
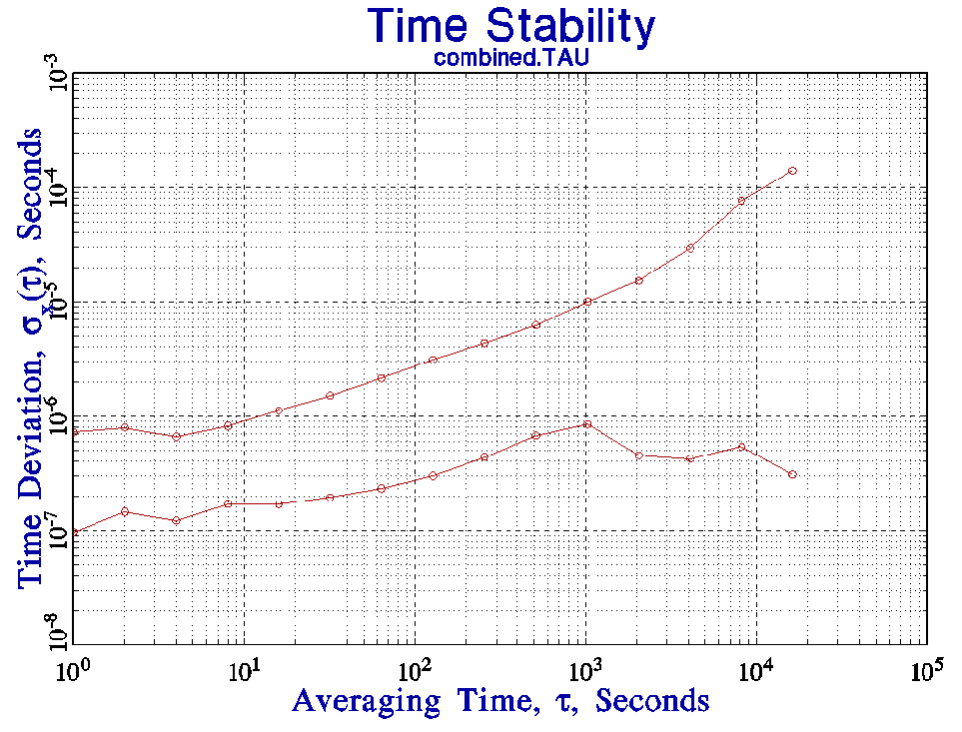
The lower curve shows the time deviation of the computer clock when it is controlled by the phase and frequency loops described in the text. The upper curve is the free-running stability as shown in Fig. 1. Note the significant improvement in the stability at longer averaging times as discussed in the text.

Figure 3 shows the TDEV of the local clock oscillator when it is synchronized by means of the combined frequency and phase loops described in the preceding paragraphs compared to the TDEV of the free-running stability of the same system. The improvement at the shortest averaging times is about a factor of 8 and is limited by the facts that there is only a weak correlation between consecutive time-difference measurements, and the average of the measured time differences is not the optimum strategy for flicker phase noise. The improvement gradually increases at longer averaging times as the assumptions of the model more closely approximate the actual statistics of the clock oscillator. In particular, note the improvement in frequency stability of nearly three orders of magnitude at the longest averaging times, resulting from the removal of the frequency variation shown in Fig. 2. No special processing is needed to remove this frequency variation; it is handled automatically by the control loop with the time constant, *k*, specified above. The data in the figure are derived from the parameters of the control loop, essentially the frequency correction computed by Eq. (5) and applied to adjust the clock frequency. The TDEV of the computer system clock is not greater than 0.8 µs for all averaging times, a value that is consistent with the TDEV of the data at the shortest averaging times. This value is consistent with the speed of the computer hardware and the jitter in the measurement of the time differences discussed above.

The previous discussion was based on a local time reference, where the measured free-running stability of the computer clock was the same as the TDEV of the time-difference measurements. However, the same algorithm can be used when the reference is a remote system and the time-difference data are acquired over a noisy communications channel such as the Internet. The method for determining sigma that was used in the previous discussion cannot be used here for a number of reasons. In the first place, the variance in the time-difference measurements arises mostly from jitter in the network channel, and this contribution must be determined for every connection because there is no universal TDEV, determined by the hardware and software of the local system, as there was in the previous discussion. In the second place, again in contrast to the previous discussion, there is no *a priori* estimate for the magnitude of the TDEV based on considerations outside of the measurement process. Finally, the outlier algorithm must function in a real-time environment, and methods that depend on a postprocessed batch analysis of a large quantity of data, such as the calculation of the median, or methods that depend on several passes through the data [18, 19] either cannot be used at all or depend on too many computer cycles to be practical. (The median and the average are not significantly different when the number of outliers is less than 1% as in the data in this study; see Fig. 4b and the following discussion.)

The considerations discussed in the previous paragraph, especially the situation wherein each communication channel is unique and must be separately characterized, mean that the parameters of the outlier detection algorithm for each channel must be derived from the data that will then be processed by the same algorithm. An outlier can be defined as a value that differs from the expected or predicted value by more than 3σ, but there is no independent, rigorously justified estimate for the value of sigma. This circular situation is not unique to this discussion. For example, the much more comprehensive method discussed in Ref. [18] has the same circular process. The values for the windowing parameters and the

threshold for defining an outlier in that study are reasonable values, but they are used because they are useful for the particular data set in the study, and there is no suggestion and no implication that they represent parameters that are globally useful. The multipass, postprocessing methods described in that study are also not appropriate for a real-time process.

The outlier process that I describe here is validated by the same considerations. The outliers are modeled as single-point time differences with no offset in time or change in frequency. Missing data is not a significant problem. This model is not presented as a universal truth, but rather as something that describes the real data that have been observed. As I will discuss below, the model is useful because the number of outliers it detects is approximately the same as the number that would be detected by a 3σ test on data that exhibited Gaussian statistics and because the synchronization algorithm that uses the time-difference data with the outliers removed in this way results in a system performance that is stable and accurate. The method does not consider changes in the frequency of the clock oscillator as outliers. These are handled by the machinery described above in Eq. (5) and illustrated in Fig. 2.

Figure 4a shows the TDEV of the time differences (after outliers have been removed) measured between a local computer system clock and a remote server when both systems are synchronized to a local reference clock as described in the previous sections. (The time-difference data that were used to compute this TDEV are shown in Fig. 4b and will be discussed below.) The time differences are measured by means of two-way message exchanges in the NTP format [6]. This format estimates the one-way delay between the client and the server as one half of the measured round-trip value, and the details of the method are described in the following text.

The message exchange between the local system and the remote server provides four time-stamp values in units of seconds: *t*_1_, the time, as measured by the local clock, when the local system sends the request to the remote server; *t*_2_, the time when this message arrives at the remote server measured by its clock; *t*_3_, the time when the remote server sends the response, again measured by its clock; and *t*_4_, the time when the reply is received by the local client, measured by its local clock. The round-trip path delay, *D*, is measured by the client as:

$D=\left(t_{4}-t_{1}\right)-(t_{3}{-t}_{2})$ (7)

where the first term on the right side is the total time that has elapsed from the time the local system sends the query to the time when it receives the response, as measured by its clock, and the second term on the right side is the delay between when the remote server received the query and when it responded, measured by its clock. The round-trip delay computed in Eq. (7) depends only on time differences, and it is therefore independent of the origin times of the local and remote systems. However, the following equations assume that the client and the server share a common timescale, which is always UTC in the current discussion. If *d* is the one-way path delay between the local system and the remote server, then the message that was sent by the local system at time t_1_ arrives at the remote server at time *t*_1_ + *d*, where these times are both measured by the clock in the local system. The time of the remote server at the instant when the message is received is *t*_2_, so the time offset of the local system with respect to the remote server is

$∆t=\left(t_{1}+d\right)-t_{2}$ (8)

If the inbound and outbound delays are assumed to be are equal, then *d* = *D*/2, and the time difference between the local clock and the remote server can be computed by combining Eq. (7) and Eq. (8).

$∆t=\frac{\left(t_{1}+t_{4}\right)}{2}-\frac{\left(t_{2}+t_{3}\right)}{2}$ (9)

This calculation is performed by the local system on every message exchange, and the computed time differences are then processed to detect outliers as described below.

The uncertainties in the measurements of the four time stamps are on the order of a few microseconds, so that the accuracy of the time-difference measurements is determined primarily by the asymmetry of the inbound and outbound delays, and not by the magnitude of the symmetric portion of the delay, which is removed by the two-way method. The stability is determined by the fluctuations in the symmetry of these delays. For example, if the true outbound delay from the local system to the remote server is not *D*/2 but *nD*, where *n* is a fraction between 0 and 1, then the assumption of the software that the one-way outbound delay is one half of the measured round-trip value will result in an error in the estimated time difference given by

$\epsilon =\left(n-0.5\right)D$ (10)

Conversely, if the local and remote systems are known to be independently synchronized by a method that is outside of the two-way measurement process, and if the measured two-way time difference between the two systems gives a value of ε, then from Eq. (10), this implies that the path asymmetry is given by

$\left(n-0.5\right)=\;\frac{\varepsilon }{D}$ (11)

The accuracy of the two-way process for measuring the time difference, and the statistics of these data are therefore directly related to the statistical properties of *n.*

The estimate of the time difference is composed of two parts. In the first part, the client system sends five requests to the remote server in NTP format as described above and receives five replies. The queries are sent as rapidly as possible, so that the time difference between the clocks in the server and the client does not change during the message exchange. These data provide five estimates of the time difference between the client and the server and the round-trip delay. If there is no correlation between the time difference and delay estimates, then the algorithm skips to the second part of the estimation process.

When a correlation between the time differences and the delay estimates is found, the client assumes that the time difference with the minimum delay estimate is most likely to be closer to the true time difference, and it corrects the other time difference estimates assuming that the other delays are larger because of path asymmetries. The sign of the correction is determined by examining the time differences estimated by using the inbound and outbound message exchanges, and it is based on the assumption that the time difference of the physical clocks is determined by the message exchange with the minimum delay. For example, if the time differences estimated by Eq. (9) change by *T* seconds between consecutive measurements, and if the measured round-trip value, *D*, changes by a very similar magnitude between the two measurements, and if the change in the time difference is greater than 3 standard deviations of the estimated free-running stability of the clock in the local system (from Fig. 1), then the change in the time difference is modeled as a change in the symmetry parameter, *n*, and not as a time step of the physical clocks, because the observed time step is not consistent with the free-running stability of the local system clock. The measurement is adjusted assuming that the minimum measured path delay is the best estimate of the true delay, where the sign of the adjustment depends on whether the change in *D* is primarily due to a change in the outbound contribution to the delay, (*t*_2_ − *t*_1_), or the inbound contribution, (*t*_4_ − *t*_3_). If the time differences between consecutive measurements differ by less than 3 standard deviations of the estimated free-running stability for an averaging time of 1 s as determined from the data shown in Fig. 1, then the time differences are accepted. The standard NTP distribution makes use of a similar method to detect changes in the asymmetry of the path delay, which is called the “huff-n-puff” filter [22].

The second part of the algorithm uses the same five-point comparison outlined above Sec. 6, except that the acceptance test is based on an extrapolation of the TDEV in Fig. 4a to 1 s, and not on the TDEV of the free-running stability as in the previous discussion. The TDEV at 1 s is estimated as 0.5 ms. The five points are examined for outliers, and the mean of the points that pass this test is used to compute the correction that would be applied to the local clock when the algorithm is used to synchronize the local clock instead of just to evaluate the procedure, as in the current discussion. Apart from the circular nature of the determination of sigma discussed above, the only difference between this configuration and the previous one discussed in Sec. 6 is that the outlier test is based on the TDEV at an averaging time of 1 s derived from Fig. 4a extrapolated back to 1 s as described above. (The TDEV for longer averaging times provides guidance in the design of the synchronization algorithm and into how well it will work, but it has no role in the process of detecting outliers.) The threshold for identifying a data point as an outlier is 3 × 0.5 ms or 1.5 ms. Figure 4b shows a small portion of the time-difference data (before the outliers were removed) that were used to compute the TDEV in Fig. 4a. The figure shows the first four outliers in 468 measurements (a fifth outlier was detected later in the data) or about 1% of the measurements, which is a typical finding. Figure 4c shows the same data when the outlier points have been removed, and the data in this time series were used to compute the results shown in Fig. 4a. The vertical scale in this figure has been expanded, but the data have not been modified apart from removing the outlier points. For comparison, the outlier fraction for data that satisfied Gaussian statistics would be about 0.26%, which is just another indication that these data cannot be characterized in that way. The figure also shows two outliers very close together in time, which is also not unusual.

**Fig. 5:**
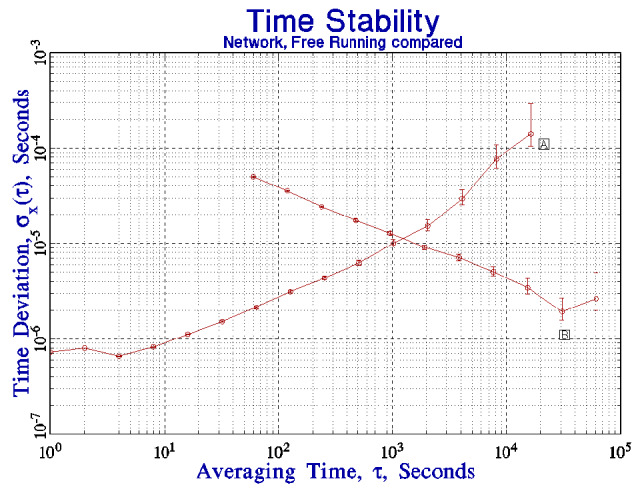
The time deviation of the link between a remote server and a client (curve B) as shown in Fig. 4a compared to the free-running stability of the clock in the client system (curve A) as shown in Fig. 1.

Figure 5 compares the time deviation shown in Fig. 4a with the time deviation of the free-running clock as shown in Fig. 1. The stability of the client system for short averaging times is limited by the jitter in the network connection to the server. The control loop cannot realize the free-running stability of the client system for this reason. The clock in the client system is more stable than the remote clock seen through the network for averaging times less than about 10^3^ s, and this determines the parameters of the synchronization algorithm. The algorithm does not apply any short-term frequency adjustments to the local clock for this reason. The adjustment process is limited to the upper end of the domain in which the time deviation of the client system is dominated by white frequency noise. From the data in Fig. 4a and Fig. 5, the interval between frequency adjustments of the local clock oscillator would be set to 10^3^ s, and the averaging parameter, *k*, in Eq. (5) would be equal to 1. A larger value of *k* is not appropriate for the data discussed here, although it might be appropriate for a network connection for which statistics could be characterized as white frequency noise over a longer region of averaging times. This favorable situation is not common on connections that use a wide-area network. The short-term stability of the time of the client system will be determined by its free-running characteristics, but the time accuracy will be limited by the time deviation of the network connection, and a stability of about 60 μs RMS would be expected at all averaging times. The time stability would improve at longer periods, as shown in Fig. 4, and the stability would be expected to be limited by the jitter in the network interrupt service delay at long times. This delay

**Figure:**
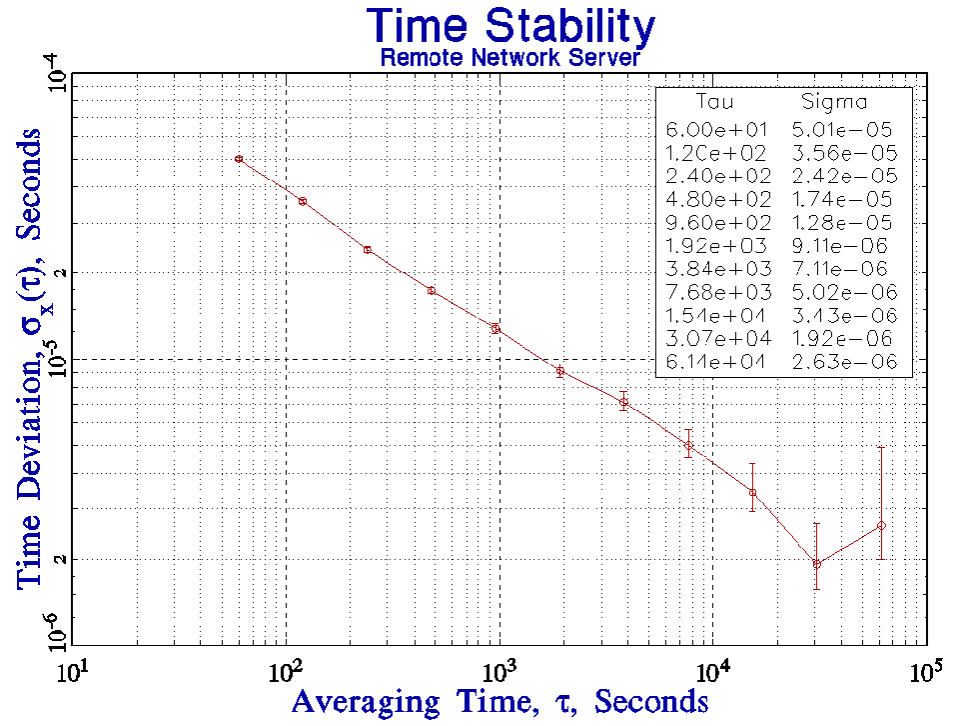
(a)

**Figure:**
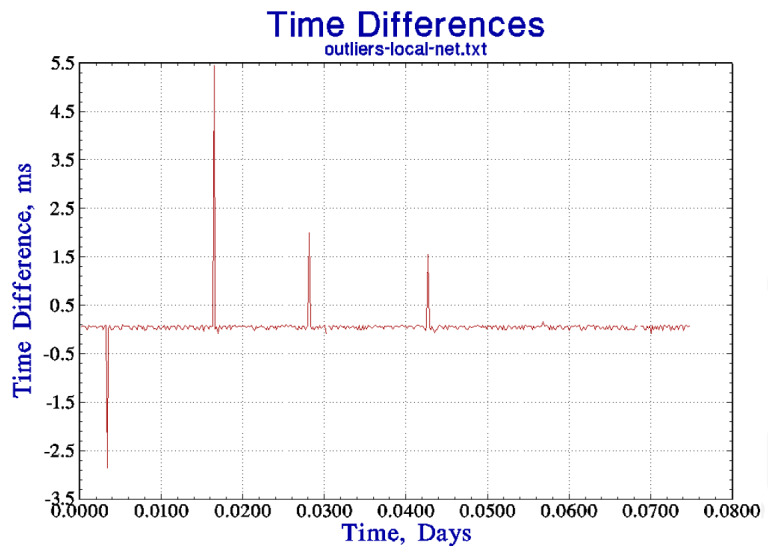
(b)

**Figure:**
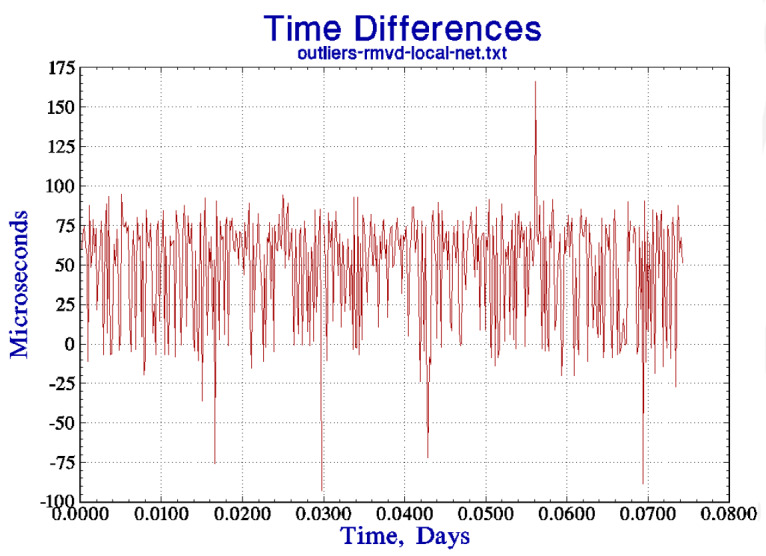
(c)

**Fig. 4:** (a) The time deviation of the link between two servers at different locations, each synchronized to a local UTC (NIST) time reference as described in the text. See also parts (b) and (c). (b) The first 468 time differences that were used to compute the TDEV in part (a) (after the outliers were removed). The outlier detection process identified a total of five time-difference measurements that exceeded the threshold of 3 times the standard deviation of the data, or 1.5 ms. The four outlier points in the first section of the data are easily identified in the figure. Also see part (c). (c) The time differences from part (b) when the outliers have been removed. Note the difference in the vertical scale between the two figures. These data are the first portion of the data used in the TDEV computation displayed in part (a).

is about 1 μs to 2 μs for the hardware at NIST, and this is consistent with the time deviation at long periods as shown in Figs. 4 and 5. Note that both in this case and in the following discussion, the stability of the combination of the local computer system clock and the remote time-difference data is better than either contribution by itself, which is a unique aspect of the algorithm. Independent measurements of the stability of the client system synchronized with this procedure confirmed these estimates; the measured TDEV agreed with the prediction within 5% for all averaging times. That is, the measured stability follows curve A in Fig. 5 for averaging times up to 1000 s, and it follows curve B for longer averaging times.

An important aspect of the algorithm described here is that it is designed to operate in the domain where the statistics of the local clock are dominated by white frequency noise, and this consideration drives the design towards a relatively long polling interval (the time between calibration requests), which is significantly longer than is often used in the standard implementation of the NTP software [16, 17]. This design insulates the local clock from the short-term jitter in the time differences measured by a typical wide-area network connection, as shown in Fig. 4a and the outliers identified in Fig. 4b. An important result of these data is that a shorter polling interval degrades the stability of the local clock, which is a counterintuitive result.

**Fig. 6:**
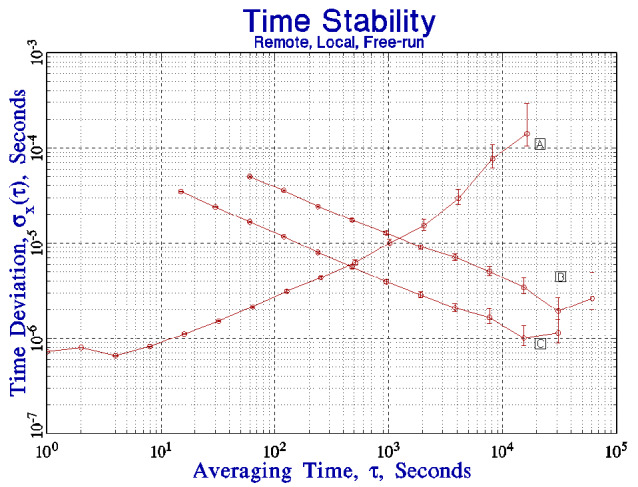
The time deviation between a client and a server when both systems are on the same local network segment (curve C) compared to the results presented in the previous figure (curve B) where the server and the client are at different locations linked by the public Internet. Curve A is the free-running stability from Fig. 1.

I can demonstrate that the time deviation of the link between the remote and local servers shown in Fig. 5 is dominated by the stability of the link. In Fig. 6, the time deviation has been added between a client and a server when both are on the same local network. The local network is not completely dedicated to this experiment; it is implemented with a single network hub and carries a small amount of local traffic. The hardware and software of the client and server systems are not changed. The time deviation has improved by a factor of 2 or 3, except at the longest periods, where the stability is limited by the diurnal frequency variations of the clock oscillators discussed above. Although both systems were located in the same laboratory for this demonstration, the clock oscillators in the two systems have somewhat different admittance to temperature variations. The general design of the synchronization loop is the same as in the remote-server case, except that the cross-over between the stability of the local free-running clock and the remote server seen through the network is at a somewhat shorter period, but the crossover is still in the domain where the local stability is dominated by the white frequency noise of the clock oscillator, and the previous discussion applies to this case as well. An important consequence of this configuration is that the cross-over between the free-running stability of the local clock and the stability of the remote server seen through the network has been pushed to shorter averaging times, so that the result is a significant improvement in the stability of the local clock oscillator for periods of a few hundred seconds. The time stability is better than 60 µs at all averaging times.

The TDEV plots for the connections to both the local and remote time servers are consistent with white phase noise at the shortest averaging times. The mean of consecutive measurements is therefore a statistically robust estimate of the time difference between the client and the server clocks, and the standard deviation of the time difference could be improved by computing the mean of consecutive measurements. However, this strategy must be used with care in a network environment. The standard deviation of the mean improves at a rate proportional to the square root of the number of measurements that are used to compute it, whereas the network load increases linearly with that number. The cost/benefit comparison is therefore unfavorable, and the increased cost in terms of network bandwidth and computer cycles *may* be a significant concern on a heavily loaded system. Even with a local network connection, where the short-term time differences can be characterized as white phase noise, the improvement in the mean of consecutive time-difference measurements is proportional only to the square root of the number of measurements that contribute to the mean. This dependence makes it difficult to realize a significant modification to the cross-over point between the mean of the time-difference measurements and the free-running stability of the local clock as shown in Fig. 6. The primary advantage to computing the mean would be to improve the detection of outlier measurements. This is an important advantage, because outliers are not rare in a networked environment. Finally, Fig. 6 illustrates the fact that the local clock is more stable than the remote reference seen through a network for short averaging times, and a control loop based on short averaging times is likely to add network noise to the local clock and degrade its stability.

This discussion illustrates the usefulness of the algorithm when the client and server systems are connected to a network that is a local network but is not necessarily dedicated to the time service. As I will discuss in the section on accuracy below, when the channel between the local system and the reference time source is implemented by a connection over the public Internet, both the accuracy of the time synchronization process and the statistics of its variation are affected more by the many routers in the path rather than by the physical length of the path itself.

## 7 Detection of Outliers

The algorithm that includes a frequency-lock loop supports the ability to detect a failure of the local reference system or a remote “false-ticker,” *i.e*., a remote reference that is transmitting the incorrect time. The algorithm compares the frequency innovation estimated in Eq. (3) with the previous frequency estimate and does not apply Eq. (5) when the innovation exceeds three times the average innovation over the preceding *k* cycles. This is useful mostly when the reference system is a remote server linked to the client over a noisy network connection. When the time reference signal is provided by local cesium clocks, the performance of these devices is monitored, and a failure of these devices is detected, by processes outside of the scope of this discussion, so that it is straightforward to identify the cause of the large frequency innovation. A failure in this situation is usually an indication of a hardware failure, and the algorithm sets the computer system clock to be unsynchronized and triggers an alarm when this happens.

It is also possible to identify a remote server that is transmitting the wrong time (without any indication that the time is not correct) by computing the time difference with respect to a different remote time server that is presumably not having the same problem. This strategy is statistically reasonable only when the expected time difference between the server and the local computer clock do not agree with the expected value within the 3σlimit. It is not appropriate to use this method in general because it increases both the network bandwidth and the number of computer cycles needed to support multiple queries on every measurement cycle with the goal of detecting the relatively rare failures of the remote reference system. The statistics of the predicted time difference of the local computer clock with respect to the remote reference should be used first.

## 8 Time Accuracy of Network Synchronization

The previous figures provided estimates of the stability of the synchronization process, but they do not provide any insight into the time accuracy. In order to address this question, Fig. 7, parts (a) and (b), shows the time difference between a client system located at JILA, formerly known as the Joint Institute for Laboratory Astrophysics, on the University of Colorado campus in Boulder, Colorado, and servers located at the three other sites operated by NIST: NIST Boulder laboratories, NIST radio station WWV near Fort Collins, Colorado, and NIST headquarters in Gaithersburg, Maryland. The displays are a running average of the measurements to attenuate the short-term white phase noise. The network links between the systems are not conditioned or special and use the public Internet; therefore, the connections are realized over the same circuits that are used to reply to time requests from all users.

The average measured one-way network delay between the sites in Boulder, Colorado, and Gaithersburg, Maryland, is about 69 ms; the one-way delay between Boulder and radio station WWV in Fort Collins, Colorado, is about 30 ms, and the one-way delay between the sites in Boulder is also about 30 ms. (Although the sites in Boulder are physically less than 1 km apart, the network path is through Denver, about 50 km away. Therefore, the network path length between the sites in Boulder is about the same as the path length between Boulder and Fort Collins, which is about 100 km north of Boulder.) The relationship between the length of these two network paths and the one-way delays is a coincidence. The network path to Gaithersburg, Maryland, is more than an order of magnitude longer, but the increase in the one-way delay is not proportionally greater.

The longer-term variations of the time differences, and especially the time offset displayed in the three data sets are in very good agreement. The peak to peak variation in the measured time differences is approximately consistent with the short-period RMS time deviation value shown in Fig. 4; the noise spectrum shows a significant flicker in the phase contribution, which is masked in Fig. 4 by the larger white phase noise component of the spectrum. (The running average process attenuates the short-term white phase noise of the data.) However, all of them show an average time offset of between −125 μs and

−150 µs between the client system and the systems at the other sites.

**Figure:**
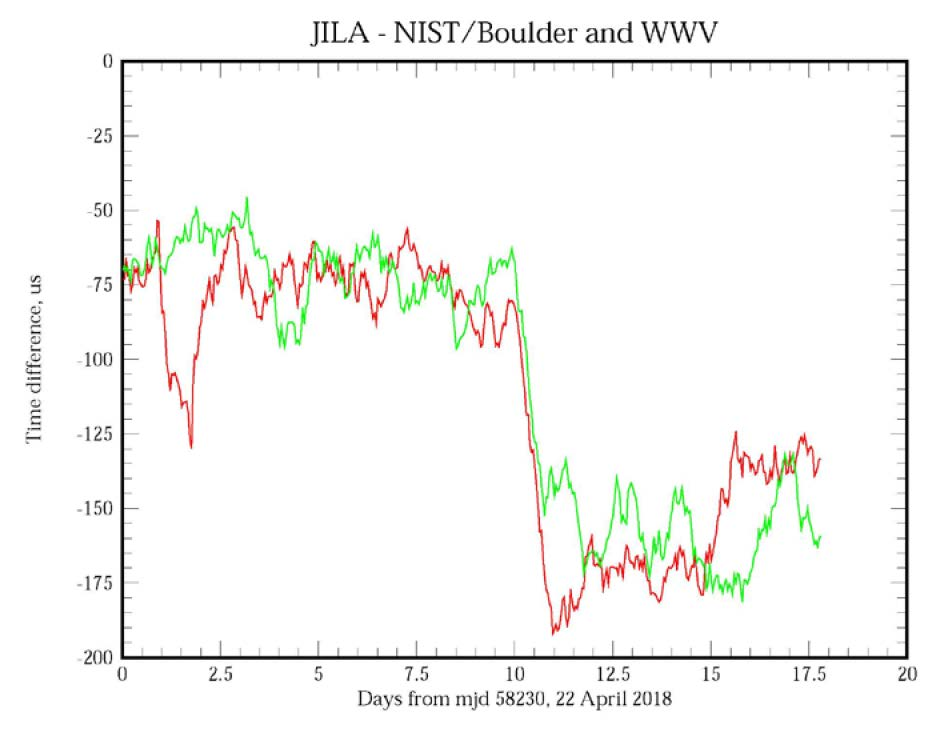
(a)

**Figure:**
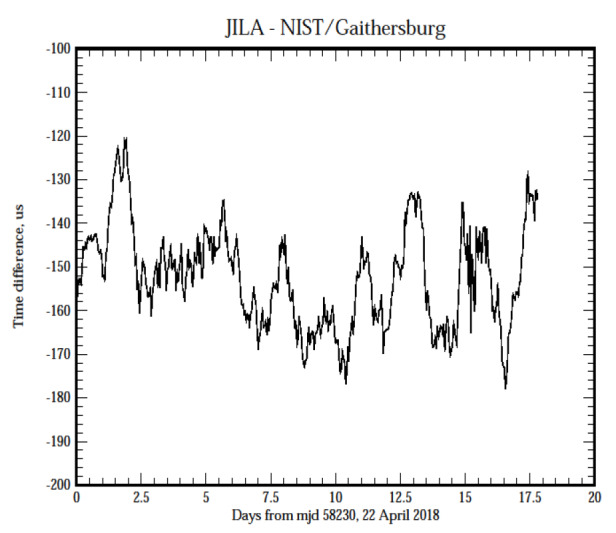
(b)

**Fig. 7:** (a) The time differences between a client system located in JILA on the campus of the University of Colorado at Boulder and servers located at NIST in Boulder (red line) and radio station WWV in Fort Collins, Colorado (green line). Both of the systems are synchronized to UTC by using the algorithm discussed in the first section of the text. The measurements have been lowpass-filtered with a running average to attenuate the short-term white phase noise of the measurements. The longer-period variations are in very good agreement, as discussed in the text. (b) The time differences between a client system located in JILA on the campus on the University of Colorado at Boulder and the server located at NIST in Gaithersburg, Maryland. Both of the systems are synchronized to UTC by using the algorithm discussed in the first section of the text. The measurements have been lowpass-filtered with a running average to attenuate the short-term white phase noise of the measurements. The static time offset is in good agreement with the value shown in part (a), as discussed in the text.

**Fig. 8:**
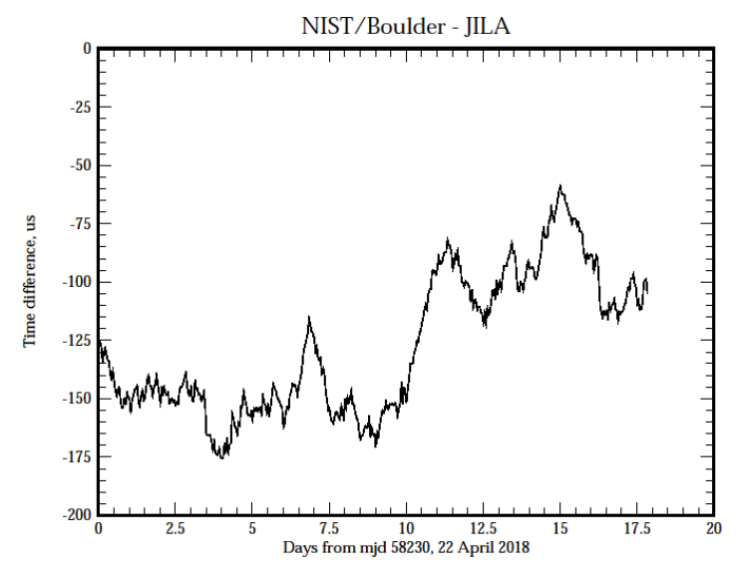
The time difference between a client system located at NIST in Boulder and a server located at JILA. The measurements have been lowpass-filtered to attenuate the short-term white phase noise. These data show the same time difference as the previous figure from the opposite perspective, *i.e*., where the system at NIST in Boulder is the client and the system at JILA is the server. Note that the time difference did not change sign when the roles of the client and server were interchanged. Also note that there is a step in the time differences starting at approximately day 10 compared to the time differences in Fig. 7a, and that this step is not present in Fig. 7b, so that the step is due to a portion of the circuit that is not common to the link to Gaithersburg.

If no other data were available, this time offset would support a robust conclusion of a time error of the client system in JILA. However, NIST operates all of the sites, and Fig. 8 shows the time difference from the opposite perspective. This figure shows the time difference when the roles of the client in JILA and the server at NIST in Boulder are interchanged. In other words, the data in Fig. 8 are the time differences between the local server at NIST in Boulder and the server at JILA as recorded by the server in NIST (Boulder) at the same time as the server in JILA was recording the data shown in Fig. 7. The times of the client and server are actually equal with an uncertainty of less than 1 μs, so that the constant portion of the measured time difference implies a static asymmetry in the network path. However, we would expect this asymmetry to be reversed when the roles of the client and server systems are interchanged, and this is not the case. (A partial reversal of the asymmetry may be present starting at about day 10 and continuing to day 12.5.) The reason is that the outbound path is chosen by the system that originates the time request, and it will not necessarily be the same as the inbound path when the query originates from the opposite direction. These data show that static asymmetries are often present in network paths, and it can be very difficult to estimate their impact on the measured time differences. The magnitude of the static asymmetry error is significantly larger than the stability estimates in Fig. 6 and is comparable to the actual time differences shown in Fig. 7a, Fig. 7b, and Fig. 8. The important conclusion is that the measured variance in the time-difference data is not necessarily a good estimate of the accuracy of the process. Our experience is that asymmetries of up to a few percent of the measured round-trip delay are quite common in long-haul public packet-switched networks, and this may limit the accuracy of network-based time transfer over the public network if no additional information about the characteristics of the network is available. For example, a time offset of 150 µs when the one-way path delay is 30 ms suggests that the path asymmetry is about 1% of the one-way delay [17, p 48], since the two-way method attenuates the impact of the asymmetry by a factor of 2. The asymmetry fraction would be smaller for the Colorado–Gaithersburg link, confirming that the asymmetry is not necessarily related to the length of the physical path between the two systems or to the one-way delay. The limitation on the accuracy caused by asymmetry in the path delay does not apply to point-to-point networks that have no network switches or routers, and it also does not apply to local-area networks. For example, the local-area-network delay presented in Fig. 6 shows no measurable time offset, and the same result is observed for other local networks within the NIST and University of Colorado facilities, even though these networks are not dedicated to the time service and carry other traffic as well. In the system considered in this study, the time differences are continuously monitored between all of the servers at all of the locations, and the maximum time asymmetry observed has been 350 µs over many months of operation.

## 9 Long-Period Fluctuations and Holdover

An important advantage of the frequency-lock loop algorithm is that it allows the characteristics of the oscillator in the client system to be described. Once the algorithm has estimated the static fractional frequency offset of the clock in the local system (3.69 × 10^−5^, about 3.2 s/d for the system described here), its time stability will be described by the free-running stability as shown in Fig. 1 if the link to the time reference is lost. The algorithm models the measured dispersion in the time-difference data as a combination of one-time outliers, which are treated as glitches and ignored, and white frequency noise of the clock oscillator. (Obviously, the glitch detector does not operate in holdover mode, since there are no measurements to analyze. The holdover performance therefore uses the most recent frequency offset that was estimated before the holdover period began, and it increases the uncertainty of that estimate by the estimate of the white frequency noise.) This is a nearly optimum strategy for averaging times out to about 10^3^ s, and measurements confirm that the RMS of the measured time differences in holdover mode agree with the predictions of the free-running stability in Fig. 1. The model is not an accurate representation of the variance in the time differences for longer averaging times, where the variance of the time-difference data is no longer dominated by white frequency noise, so that the average frequency is no longer a statistically optimum estimator, but it is possible to extend the model to address this deficiency.

.

The first problem is that the method used by the model to estimate the deterministic frequency offset of the oscillator in the presence of white frequency fluctuations involves averaging the frequency estimates over some time interval of order *T*_max_ (10^3^ s in the current discussion) because fluctuations with shorter periods are taken to be white frequency noise. The result is that the frequency estimates displayed in Fig. 2 lag the true frequency variation. The frequency steps in the figure are detected with a time delay of order *T*_max_ s, so that the time stability in holdover mode is degraded. (Time-difference data, which could detect the frequency step more rapidly, are not available in holdover mode.) Since the frequency variation in Fig. 2 has a strongly periodic component, a first-order correction to the frequency estimated by Eq. (5) is to feed-forward (that is, to anticipate) the diurnal frequency variations estimated from the measured diurnal variation in the preceding 2 d. For example, the average step in frequency that was estimated near 0 hours UTC on the previous 2 d is administratively applied to the clock oscillator at that time today, even though there are no measurements to confirm that step when the algorithm is operating in holdover.

The frequency variation displayed in Fig. 2 is not purely diurnal, and the choice of a 2 d averaging window is an administrative choice that works well with the data. I validated the choice of a 2 d estimate of the diurnal contribution to the frequency variation by testing various choices on measurements of free-running time differences. However, this averaging time is almost certainly not a universal constant, and the optimum choice may be different for different temperature environments. The algorithm addresses this variability in these quasi-periodic contributions to the variance of the time differences by periodically reevaluating the averaging time constant, *k*, in Eq. (5), as I discussed above. In general, the success (or lack of success) of any feed-forward algorithm depends on the past being a robust estimator of the future. Based on a limited data set, these algorithms can predict about 60% of the variance in the general case, and a much larger fraction of the variance when the driving term has a very stable autocorrelation for some time lag, as is the case for the data in this study and for any configuration where a “bright line” in the Fourier expansion of the time-difference data makes a significant contribution to the variance.

**Fig. 9:**
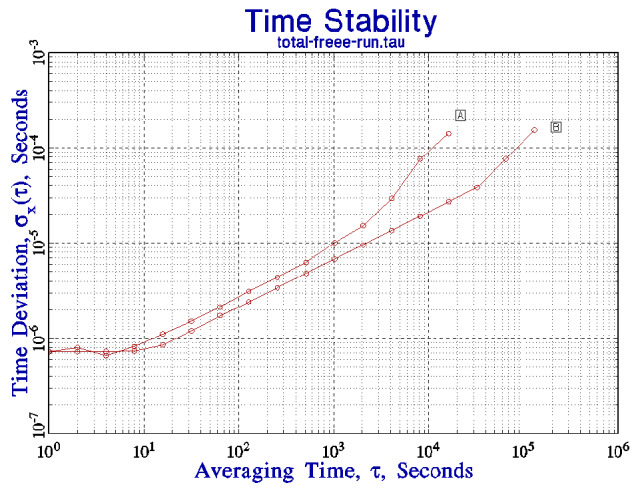
The time deviation of the full model, including the feed-forward of the diurnal variation (curve B) and the estimate of the longer-term frequency drift compared to the initial free-running stability as shown in Fig. 1 (curve A). The feed-forward process anticipates the changes in the clock frequency shown in Fig. 2 by applying a frequency adjustment based on the variation observed on the previous 2 d at the same time.

When the updated holdover model incorporates the nearly diurnal component of the frequency variance, the remaining residual variation in the frequency shows a frequency drift that is approximately linear over the period of a few days. The magnitude of the drift in fractional frequency is approximately 1.5 × 10^−8^ d^−1^ (1.75 × 10^−13^ s^−1^), or about 5% of the diurnal contribution. If the free-running stability at longer periods were due totally to this frequency drift, it would contribute about 0.7 ms to the time dispersion, which is in reasonable agreement with the extrapolation of the free-running stability to an averaging time of 1 d. The measured time dispersion is about 1.2 ms for an averaging time of 1 d, which is reasonable given that the frequency drift is only approximately linear, and the diurnal frequency variation does not exactly average to zero over a 1 d period. When this linear term is added to the feed-forward component of the model, the TDEV of the complete model is shown in Fig. 9. The TDEV is compared to the original free-running stability taken from Fig. 1. Although there is some improvement at short averaging times, the primary impact of these additional terms is at longer averaging times. The additional terms extend the domain in which the white frequency noise of the oscillator is the dominant contribution to the measured variance. The holdover performance at an averaging time of 16 384 s is improved by a factor of about 5.

At periods longer than 16 384 s, the variance is increasingly dominated by nonstationary effects, which probably cannot be modeled with any accuracy by a universal model. However, the holdover stability of any system will probably be better than 0.5 ms for averaging times out to at least 1 d. Both the stability and accuracy of the time of the computer system clock will return to the values that characterize the performance of the control loop when the reference data become available again, as described in the previous sections and as shown in Fig. 3, Fig. 6, and Fig. 7. The time required to return to this level of performance depends on the length of the holdover period, but it will be significantly shorter than the maximum time of 260 s that is required for a complete cold start.

## 10 Leap Seconds

An extra “leap” second is added to Coordinated Universal time (UTC) at irregular intervals so that the magnitude of the difference between UTC, which is the reference timescale for the clock synchronization algorithm, and UT1, a timescale based on the position of Earth in space, is maintained less than 0.9 s [23]. When a leap second is needed, it is added following 23:59:59 UTC, usually on the last day of June or December [23, 24]. The official name of the leap second is 23:59:60, but that time value cannot be represented in the format that I described in Sec. 3. Therefore, the computer system clock implements the leap second by repeating the time value corresponding to 23:59:59 a second time, and the subsequent second has the time tag corresponding to 00:00:00 of the next day. The time stamps effectively “forget” the interval of the extra second once it has occurred. The repetition of the time value corresponding to 23:59:59 also introduces an ambiguity in the ordering of time stamps, since 23:59:59.1 (the second time) actually occurred *after* 23:59:59.2 (the first time).

When a measurement interval includes a leap-second event, the frequency estimated by Eq. (3) will be too large because the actual elapsed time between the two time-difference measurements is *T*_min_ + 1 and not *T*_min_. The fractional frequency error will be of order 1/*T*_min_, which is about 10% at the 10 s averaging time used in the previous example. Even if the averaging time is closer to *T*_max_, this effect is almost always too large to be ignored, since the offset frequency is generally of order 10^−5^, and the estimated variations in this frequency are generally two orders of magnitude smaller. See Fig. 2 for an example.

The simplest method of addressing this problem is to postpone a calibration cycle when a leap second event is imminent, and the current version of the algorithm uses this method. It would also be possible to explicitly correct the frequency estimated by Eq. (3).

The message formats that are used by NTP and many other common network message formats have no means of distinguishing between the first and second transmissions of the value corresponding to the time of 23:59:59 on a leap-second day. A client system that uses this type of message exchange generally must postpone the calibration cycle when this time tag is received on a leap-second day.

## 11 Summary and Conclusions

I have described the design of an algorithm that will synchronize the clock in an unmodified general-purpose computer. The design of the synchronization algorithm is based on the statistics of the oscillator in the client system and the stability of the channel that provides the reference time. When the reference time messages are received from a remote system over a relatively unstable network connection, the characteristics of the channel make a significant contribution to the performance of the synchronization algorithm, and the characteristics of the remote clock are less important. In all configurations, the design of the algorithm combines the reference data received from some external source with the free-running statistics of the local clock oscillator to realize a stability that is better than either contribution by itself. In this respect, it is different from, and an improvement on, other algorithms that are fundamentally designed around a master-slave relationship, which does not make optimum use of the statistics of the local clock oscillator. This advantage is particularly useful when the reference time signals are received over a noisy, unstable network connection; the algorithm provides the optimum cross-over point between the stability of the local clock and the stability of the remote reference as seen through the network. Simple synchronization algorithms, based on phase-locked loops, possibly combined with a master-slave configuration, are far from optimum in this situation because the local clock is more stable at short averaging times than the remote clock seen through the network and because the remote data cannot be characterized as simple white phase noise for which the variance can be robustly attenuated by averaging.

Network-based synchronization algorithms use the two-way protocol to estimate the transmission delay between the reference source and the client system. This method assumes that the one-way delay is one half of the measured round-trip value, and the accuracy of this assumption depends on the equality of the inbound and outbound delays. Any delay that is outside of the measurement loop is not estimated at all, so that asymmetries in such delays must be minimized by careful software design. (Delays in the client and server software are often of this type.) Static asymmetries of up to a few percent of the measured round-trip delay are often observed, and these asymmetries introduce systematic errors into the estimated time differences. Figures 7 and 8 demonstrate an example of a measured static asymmetry of about 150 μs, which is about 1% of the shortest one-way channel delay of the data in the figure. There is nothing special about the links between the servers that were used to acquire the data in these figures. The same network connections were used to support the general public access to the time servers. The figure also demonstrates that this static asymmetry is not always directly proportional, either to the channel delay or to the physical distance between the client and the server systems, and it depends on the detailed configuration of the link between the server and the client systems. This demonstration depends on the fact that all of the systems in the time comparisons are independently synchronized with an uncertainty that is negligibly small on the scale of the figures. Unfortunately, it can be very difficult to estimate the magnitudes of these static systematic offsets in the general situation, and they are a fundamental limit to the *accuracy* of time transfer over a wide-area packet-switched network. However, the algorithm described here mitigates the impact of variability in the symmetry of the network delay by incorporating the statistics of the local clock in an optimum manner, by providing an objective method for detecting and removing outliers, by providing a statistically robust model for maintaining the synchronization of the local clock in a holdover situation when the reference data are not available and especially in the presence of quasi-periodic perturbations to the frequency of the clock oscillator, and by providing a platform for responding to the longer-term variability in the characteristics of the frequency of the local clock oscillator and the jitter in the network delay. The limitation on the accuracy of the synchronization process is much less serious over local-area networks, but many of the other problems remain, except in the very unusual situation of a channel that is dedicated to time synchronization, carries no other traffic, and has no routers, switches, or other network elements in the path. These limitations are independent of the details of the format that is used to exchange time messages and will not be improved by simply replacing the NTP message exchange used in this discussion with some other format. Their impact can be significantly reduced if the communication link is implemented with circuits that have a more symmetric delay. Therefore, the algorithm is a very useful adjunct to a local internal network that distributes time information from a GPS receiver to a number of client systems, even when this network carries other traffic in addition to the timing information.

**12. List of Variables**
*D*: The round-trip path delay measured by the two-way message exchange.
*ε*: The time error in a two-way message exchange caused by an asymmetry in the inbound-outbound path delays.
*k*: The dimensionless constant that is used in the exponential filter to attenuate the white frequency noise of the clock.
*n*: The asymmetry parameter in the two-way message exchange. The outbound fraction of the round-trip delay.
*t*_1_, *t*_2_, *t*_3_, *t*_4_: The time stamps used as part of the two-way message exchange.
*T*: The averaging time (measured in seconds) that is used to estimate the offset frequency of a clock based on consecutive, measured time differences
*T*_c_: The time constant (measured in seconds) for a control loop that is critically damped.
*T*_min_, *T*_max_: The lower and upper averaging times (measured in seconds), respectively, for which the variation in the measured time differences can be characterized as white frequency noise.
*x*: The time difference between two clocks (measured in seconds). In the current discussion, the time difference is measured between a device under test and a second device that is much more stable and much more accurate than the device under test, so that it can be considered as perfectly stable and accurate. The characteristics of the time difference are driven by the statistics of the device under test, the measurement process, and the channel that links it to the reference device.
*y(t)*: The fractional frequency offset of a clock at time *t*, measured as the time dispersion over some interval (measured in seconds) divided by the interval (also measured in seconds). The quantity has no dimensions. The time, *t*, is in units of seconds from some arbitrary origin. The frequency estimate is based on the time differences measured at two different epochs separated by the averaging time in the denominator, so that the absolute time origin is not important. This fractional frequency has no connection to the frequency of the internal clock oscillator, which is not used in the current discussion.
$\overset{-}{y}(t)$: value of *y*(*t*) smoothed by the exponential filter with time constant *k*. The parameter has no dimensions.
*δx*: The uncertainty in the measured value of x or the dispersion of consecutive time-difference measurements. The units are seconds.
*δf*: The fractional frequency offset of a clock, estimated as the ratio of the measured time difference to the averaging time. The quantity has no dimensions.
*σ_x_*(*τ*): The time deviation (TDEV) of a clock as a function of the averaging time, τ. Both TDEV and the averaging time are in units of seconds. The definition and the importance of this parameter are discussed in the Appendix.

## 13 Appendix: An Introduction to Clock Statistics

In the time and frequency literature, the time of a clock, *x*, is measured in seconds and is the time *difference* between the device under test and a second device. In the discussion in the main text, both the accuracy and the stability of the second device are much better than the corresponding parameters of the device under test, so that the variations in these data are due solely to the variation in the device under test, the channel that links it to the reference device, and the measurement process.

The frequency of a clock, *y*, is the evolution of the time over some averaging interval, τ. Thus

$y\left(t\right)=\frac{x\left(t\right)-x(t-\tau )}{\tau }$ (A1)

The numerator and denominator of Eq. (A1) are both in units of seconds, so that the frequency is a dimensionless quantity. This frequency has no connection to the physical frequency of the clock oscillator. Some analyses also use a frequency drift parameter, defined as the evolution of the frequency over some time interval. Thus,

$d\left(t\right)=\frac{y\left(t\right)-y(t-\tau )}{\tau }$ (A2)

The frequency drift has units of s^−1^. The frequency drift is often referred to as frequency *aging*, although aging often implies a constant, quasi-deterministic value, whereas drift is not limited in this way. The parameters *y* and *d* are the *average* frequency and frequency drift, respectively, over the averaging time, τ. In terms of these parameters, the time of a clock at some epoch *t* seconds after some origin epoch is estimated by

$x\left(t\right)=x_{0}+yt+\;\frac{1}{2}dt^{2}$ (A3)

where *x*_0_ is the time difference at epoch *t* = 0. The three parameters *x*, *y*, and *d* are related to the characteristics of the physical clock hardware, but they are generally not known *a priori* and must be estimated from the data. The parameters *y* and *d* are not constant for real clocks, so that Eq. (A3) is usually expressed in an iterative form, which relates the current estimates of parameters *x*, *y*, and *d* to the estimates computed on the previous measurement cycle:

$x\left(t\right)=x\left(t-\tau \right)+y\left(t-\tau \right)\tau +\frac{1}{2}d\left(t-\tau \right)\tau^{2}+\;\xi$ (A4)

where ξ is a noise contribution to the time difference. The frequency and frequency drift parameters evolve according to

$y\left(t\right)=y\left(t-\tau \right)+d(t-\tau )\tau +\;\eta$ (A5)

$d\left(t\right)=d\left(t-\tau \right)+\;\zeta$ (A6)

The parameters η and ζ are noise contributions to the frequency and frequency drift terms, respectively. The discussion in the main text ignores the drift term, which is generally valid for quartz-crystal oscillators provided the time interval between data points satisfies

ζτ≪η (A7)

which effectively pushes the contribution of the drift term into the variance of the frequency. The stochastic frequency variation, η, will have a nonzero mean if the frequency of the oscillator has a statistically significant, nonzero frequency drift, and the simplification of the following equations is not suitable for this situation. The condition in Eq. (A7) also limits the usefulness of adding additional terms, with higher-order dependence on τ, to the model of the time differences in Eq. (A4), since it would require increasingly longer values of τ to separate the impacts of these terms from the contribution of the frequency noise amplitude, η. These considerations are generally not important for the quartz-crystal oscillators typically used in computer hardware and the time intervals between measurements discussed in the main text. With this simplification, Eq. (A4) and Eq. (A5) become

$x\left(t\right)=x\left(t-\tau \right)+y\left(t-\tau \right)\tau +\xi$ (A8)

$y\left(t\right)=y\left(t-\tau \right)+\eta$ (A9)

It is easiest to work with a constant time interval, τ, between data points, but this is not a requirement of the model.

The separation of the time difference into the deterministic parameters *x* and *y* and the stochastic parameters *ξ* and *η* is not unique, because the deterministic parameters are not strictly constant values. In general terms, a deterministic parameter has a variation that is not significant over the measurement interval, τ, whereas this limitation does not apply to the stochastic parameters. For example, the frequency value that is used in Eq. (A8) is assumed to be a constant over the time interval *t* − *τ* to *t*, even though Eq. (A9) indicates that this is only an approximation, which is reasonable only if *y*(*t*−*τ*) >> η. This restriction is easily satisfied in the discussion in the main text, where the stochastic frequency variation is 1% or less of the deterministic estimate. This restriction and Eq. (A7) limit the range of values of τ, the interval between measurements. The evolution of the parameters *x* and *y* in Eq. (A8) and Eq. (A9) is effectively averaged over the time interval τ. The model does not describe the detailed performance of the clock at shorter time intervals.

In the discussion in the main text, the only observable is the time difference, *X*(*t*), and the model parameters in Eq. (A8) and Eq. (A9) must be deduced from this single data set by comparing the measured time difference, *X*(*t*), with the prediction of the model, *x*(*t*). The are more parameters than observations, so that there is no unique solution. From the perspective of the discussion in the main text, the most important parameter is the frequency offset, *y*, because it is the primary parameter used to steer the computer clock between measurements of the time difference, *X*, so as to drive *X* to zero. Incorporating glitch detection into the algorithm is very important for this reason. A time error will have a much more serious impact if it is not detected and pulls the frequency estimator.

The recursive nature of the model in Eq. (A8) and Eq. (A9) means that the time and frequency at any epoch are the integral of the previous values back to the origin epoch. In the Fourier domain, this integration scales the contribution of the frequency noise to the variance of the time by a factor of 1/*F*^2^, where *F* is the Fourier frequency argument of the power spectral decomposition of *y*(*t).* Thus, even when the frequency noise term, η, can be approximated as a simple “white noise” variable with a well-defined mean and standard deviation, the statistics of the time are no longer “white,” but instead have a “red” spectrum, with a large component of the power spectral density at very low Fourier frequencies. By the same argument, the Fourier amplitude of the contribution of the stochastic frequency drift to the time is scaled by a factor of 1/*F*^4^.

This increase in the power spectral density at very low Fourier frequencies has two consequences. (1) Although a Fourier decomposition expands the variance in a complete set of basis functions in principle, a large part of the power is concentrated at the very lowest Fourier frequencies, making a detailed analysis difficult in the Fourier domain. (2) Since the variance of the time is not a simple random variable, the standard statistical parameters of the mean and the standard deviation are not appropriate. They are not statistically robust, even though they always exist in a formal sense.

This simple analysis suggests that the Fourier decomposition of the variance of the time data can be approximated by three terms: a “white noise” contribution driven by ξ, which is constant at all Fourier frequencies and therefore has a power spectral density function proportional to *F*^0^, and two “red” contributions that scale as 1/*F*^2^ and 1/*F*^4^, respectively, relative to the white-noise contribution. Experience with real clock data shows that there are also significant contributions to the Fourier decomposition of the variance that scale as 1/*F* and 1/*F*^3^. In the Fourier domain, the variance of the time data can be approximated by five terms with power spectral densities varying as *F^j^*, where *j* takes on integer values between 0 and −4. The first three terms, with exponents 0, −2, and −4, arise from “white phase noise,” “white frequency noise,” and “white frequency drift noise” (which is usually called “random walk frequency noise” in the literature), respectively, while the two additional terms, with exponents −1 and −3, are identified as “flicker of phase” and “flicker of frequency” contributions to the time, respectively. (The infinite total power implied by a white spectrum over *all* frequencies, and the divergence at the lowest Fourier frequencies implied by the previous discussion are not a problem for real data, because real data are band-limited in the Fourier domain by the total length of the data set at the lower end and by the time interval between measurements at the upper end. There are corresponding limitations on the analysis in the time domain, presented below.)

The goal of the statistical analysis is to identify the magnitudes of each of these contributions. Since each of these contributions affects the model of the time, *x*, with a coefficient that is a different function of the averaging time, *τ*, it is usually possible to find regions of averaging times where only one of these terms makes a significant contribution to the variance. This is important because the optimum strategy for mitigating the contribution of the noise depends on knowing the noise type. As a simple example of this point, it would not be optimum to adjust the frequency of the clock oscillator in a domain where the dominant contribution to the variance of the time data was white phase noise driven by ξ because the observed variance does not arise from fluctuations in the underlying frequency of the oscillator. To the extent that ξ is modeled as a random variable with Gaussian statistics, the optimum strategy is to attenuate the variation in the *time differences* by averaging them. On the other hand, when the dominant noise type can be characterized as white frequency noise, the optimum strategy will be to average consecutive *frequency estimates*. The time differences, which are the integral of these frequency fluctuations, no longer have a spectrum that is even approximately Gaussian, so that neither the mean time difference nor its standard deviation is a statistically robust quantity in this situation, even though both exist in a formal sense. Unfortunately, there is no statistically optimum method for addressing noise processes in the flicker domain, although approximate strategies have been discussed [25, 26]. The algorithm in the main text uses the conventional mean of the data in the flicker domain as a secondary contribution to the adjustment process. The estimates resulting from this approximation are very close to the results of the other strategies, which are not used because they are much more expensive from the computational perspective without any corresponding increase in the accuracy of the prediction of the future time difference based on the currently available data.

The Allan variance [27, 28] is a time-domain estimator designed to address the difficulty of an analysis in the Fourier domain that is a result of the strong divergence of the power spectral density of *x* at low Fourier frequencies. There are a number of different versions of this variance, but the form that is used in the current text is TDEV [29], the time deviation, σ*_x_*(τ), which is the square root of the time variance and is based on the modified Allan variance [29]. This variance is calculated by computing the average of the square of the second difference of the time measurements:

${\sigma_{x}}^{2}\left(m\tau_{0}\right)=\frac{1}{6m^{2}(N-3m+1)}\sum_{j=1}^{N-3m+1}{\left\{\sum_{i=j}^{j+m-1}\left(x_{i+2m}-2x_{i+m}+x_{i}\right)\right\}}^{2}$ (A10)

where *x_k_* is the time at epoch *t_k_*. The time interval between points is τ_0_ = *t_k_* − *t_k_*_−1_, which is a constant value. The variance in Eq. (A10) is usually calculated for averaging times with *m* = 1, 2, 4, ….

The TDEV statistic provides an estimate of the variance of a time measurement based on previous data. However, its most important use is that the slope of a log-log plot of σ*_x_*(τ) as a function of τ can be used to determine the dominant noise type and therefore the optimum strategy for mitigating the impact of the noise contribution. Table 1 shows this relationship.

**Table 1:** The relationship between the noise type and the slope of a log-log plot of TDEV.

Noise Type	Exponent of Power Spectral Density	Slope of Log-Log Plot of TDEV
White Phase Noise	0	−1/2
Flicker Phase Noise	−1	0
White Frequency Noise	−2	1/2
Flicker Frequency Noise	−3	1
Random Walk Frequency Noise	−4	3/2

The TDEV statistic is particularly useful in the current discussion because it can distinguish between white phase noise and flicker phase noise, whereas the simpler Allan variance does not do this.

The optimum strategy for mitigating the contribution of the noise depends on its type. The mean and standard deviation are robust statistical estimators when the underlying noise type has a white spectrum. Thus, averaging the time/phase data is optimum when the noise type is white phase noise, whereas averaging the frequency is optimum when the noise type is white frequency noise. Although these averages always exist in a formal sense, they are statistically optimum only in the corresponding “white” domain. They must be used with care outside of the domains where they are applicable because they are neither stationary nor statistically robust.

For example, Fig. 1 in the main text shows the TDEV of a free-running computer clock. Based on the discussion in this section, there is *no* domain in which the variance of the time-difference data can be modeled as white phase noise, so that a control loop based on the average of the time data alone can *never* be statistically optimum. The primary control loop in the main text operates on the mean frequency, where that parameter is estimated in the domain where the dominant contribution to the variance is white frequency noise. In general, there are no optimum strategies for mitigating flicker noise, although the mean time difference can be used with the understanding that it is not a statistically robust quantity. Therefore, the mean time difference is used only as an adjunct to the primary control, which operates in the white frequency noise domain.
